# “You are so ugly, you whore”- girls in rural Sweden discuss and address gendered violence

**DOI:** 10.1080/17482631.2019.1695308

**Published:** 2019-12-12

**Authors:** Lotta Brännström, Sara Nyhlén, Katja Gillander Gådin

**Affiliations:** a Public Health, Department of Health Sciences, Mid Sweden University, Sundsvall, Sweden; b Department of Humanities and Social Sciences, Mid Sweden University. Mid Sweden University, Sundsvall, Sweden

**Keywords:** Hegemonic masculinity, performativity, sexual violence, sexual harassment, adolescence, gender norms

## Abstract

Girls face gendered violence on an everyday basis, and this may have severe health consequences. **Purpose:** The aim of this study was to learn about gendered violence facing girls in rural Sweden in their everyday life, as it is experienced by the girls themselves. **Method:** Using the photovoice method, we worked with 35 girls in an upper secondary school, aged between 16 and 20, to explore how they navigated social spaces and developed strategies for increased safety. **Results:** Thematic analysis revealed two main themes (*constant fear* and *strategies*) and four sub themes (*fear of being raped, fear of being labelled and excluded, being “appropriately” sexually active*, and *appearance and performance for increased feelings of safety*). **Conclusion:** We considered how gendered violence facing girls led to fear and marginalization in a range of situations and interactions. Consequently, girls occupied significantly smaller social spaces compared to boys, and we argue that this was reproduced and upheld through everyday practices informed by hegemonic masculinity and performativity.

## Introduction

Violence against women is a global public health problem of epidemic proportions that penetrates all parts of society and knows no cultural or economic limitations (Ellsberg et al., 2015) Gendered violence is defined as “any act of gender-based violence that results in, or is likely to result in, physical, sexual or mental harm or suffering to women, including threats of such acts, coercion or arbitrary deprivation of liberty, whether occurring in public or in private life” (United Nations, 1994). According to the World Health Organization (WHO) estimations (World Health Organization, 2017) one in three women globally have experienced gendered violence, which is both a cause and an effect of social inequalities (Krug, Mercy, Dahlberg, & Zwi, 2002). This violence is not only facing adult women but also girls and young women. According to international studies, both gendered violence and social inequalities have serious health effects. Gendered violence against girls and young women limits access to healthcare and education, and affects the spread of STDs and HIV (Dellar, Dlamini, & Karim, 2015; Moletsane, Mitchell, & Lewin, 2010). It is associated with deliberate self-harm (Landstedt & Gillander Gådin, 2011) depression, poor psychological and physical health, and increased risk of alcohol and substance misuse (Bucchianeri, Eisenberg, Wall, Piran, & Neumark-Sztainer, 2014).

### Gendered violence against girls and young women in Sweden

Sweden is internationally known as a global leader on gender equality (Heikkilä, 2016; Magnusson, Rönnblom, & Silius, 2008; Sandberg, 2013). However, recent reports show that approximately one-third of Swedish women do not feel safe outside alone late at night, and some women avoid going out after a certain time because they fear experiencing violence (Axell, 2018). This fear is not unfounded, given that evidence presented by (European Union Agency for Fundamental Rights, 2014) which suggests that Sweden has a significantly higher rate of physical, sexual, and psychological violence than the European Union (EU) average. In Sweden, 46% of girls and women over the age of 15 report that they have experienced physical and/or sexual violence, compared to 33% of EU citizens. Also, 9% of girls and young women in Sweden between the ages of 16 and 24 report that they have experienced sexual violence (Command et al., 2017). If Sweden’s progress towards gender equality is measured by equal representation in institutions and employment, misleading perceptions may result, since this offers only a partial picture that obscures structural inequalities and gender systems (Lister, 2009; Pease, 2015).

While statistics indicate progress towards equality on a macro level, the vulnerability of women and girls on a micro level may in fact be masked by these statistics. Furthermore, existing studies on gendered violence and fear of violence among Scandinavian women and girls mainly focus on urban contexts (van der Burgt, 2015) especially in relation to urban planning (Dahl & Henriksson, 2010). This leaves a gap in the research covering violence against young women in rural areas (within social contexts outside of intimate partner relationships). There is a need to understand gendered violence in the realm of public spaces and social contexts from the perspective of girls and young women. This study examines violence perpetrated by boys against girls from a heteronormative position. Therefore, same-sex violence or violence perpetrated by girls against boys is not considered here.

## Theoretical framework

### Performativity and hegemonic masculinity

West and Zimmerman (1987) introduced the notion of gender as something people accomplish through daily practices by “doing gender”. This concept refers to behaviour and interactions that establish masculine or feminine activities. It involves viewing gender as something we do, rather than something we are (West & Zimmerman, 1987). Butler’s notion of performativity has similarities to “doing gender” but emphasizes that the individual act is done because of gender and argues that “gender is the mechanism by which notions of masculine and feminine are produced and naturalized’’ (Butler, 2004, p. 42). Butler explains gender as something we relate to partly on the foundation of categories as “girl”, “boy”, “woman”, or “man”, and partly through the heterosexual matrix, and it is through these categories that we perform and position ourselves and others in different contexts (Butler, 2004). Looking at gender as performativity and social constructs, based on heteronormative values anchored in bodily practices, reveals patterns of dominance and subordination. These patterns of performativity might lead to boys and young men having distorted values of how to treat girls and young women (Butler, 1990; Connell & Messerschmidt, 2005) and also influence how girls and young women position themselves.

Gendered violence largely merges with performances of hegemonic masculinity (Robinson, 2005). Therefore, we can also apply the concept of hegemonic masculinity to understand structures that influence attitudes among boys and young men, as well as in relation to and among girls and young women. Hegemonic masculinity has a direct relationship with gendered violence (Gruber & Fineran, 2016), in that it places heterosexuality and an idealized notion of masculinity as a point of departure. It not only assumes masculinity as being cisgendered and heterosexual, but also demands a set of practices that position men as active (Beasley, 2015; Connell & Messerschmidt, 2005). The concept of hegemonic masculinity can be used to understand violence against girls and young women as a way to construct and reinforce heterosexual masculinities and the linking of gender norms to power dynamics to ensure the dominant position of men over women (Connell & Messerschmidt, 2005; Jewkes et al., 2015). Gendered violence has a profound impact on girls’ lives, and we need to know more in order to better address the problem and work for gender equality. The aim of this study is to learn about gendered violence facing girls in their everyday life in rural Sweden, as experienced by the girls themselves.

## Methodology

### Photovoice

The study is interactional and departs from the girls’ positions and experiences combined with the idea that knowledge develops through participation (Mitchell & Sommer, 2016; Wang, 1999; Wang & Burris, 1997). To effectively access girls’ views and reflections on society and to ensure meaningful participation, we used photovoice, which is a form of participatory action research. Photovoice has been found to be especially useful in work that seeks to acknowledge the voices of girls and young women in relation to critical social and health issues in their lives (Mitchell & Sommer, 2016; Wang, 1999; Wang & Burris, 1994, 1997). Photovoice is different from other qualitative methods in its ability to disrupt common hierarchies between “experts” and informants, and to merge participants and researchers (Gubrium & Harper, 2013). We combined photovoice with workshops and group interviews, as the process of discussing images together with participants to reach a deeper understanding of what is being ’said’ is one of the key aspects of photovoice. As researchers, we need the girls’ explanations to avoid attaching unintended meanings to what we see (Mitchell, 2011; Mitchell, De Lange, & Moletsane, 2017; Tinkler, 2013). This is partly to address the aspect of positionality (because the understanding of a situation might be tainted by one’s position) and partly because if and when respondents use symbolic images to tell a story, such as a staircase or a door, we might need an explanation to understand the meaning.

### Research context and participants

The study was conducted in a rural upper secondary school in the northern part of Sweden. We invited learners from one vocational program, two theoretical programs, and newly arrived migrant girls who attended a special language program. Photovoice and research questions were embedded in the ordinary curriculum for the theoretical and vocational program, so project tasks were mandatory for students registered for those programs, but participation in the research project was voluntary. Students who chose to abstain from participation in the research were put into a group of their own and from this group, none of the photographs were included in any research material and workshops were not audio recorded. The research project involved 35 girls, five of whom were newly arrived migrants. Participants’ ages ranged between 16 and 20. They were divided into five different groups, based on which education program they were enrolled in. Based on the discussions and topics/situations that the girls addressed during the study, we used the biological categorization of boys and girls and approach the study from a heteronormative standpoint. However, we acknowledge that there are variations in gender identities and sexual orientations.

### Process and procedure

**Figure 1.**
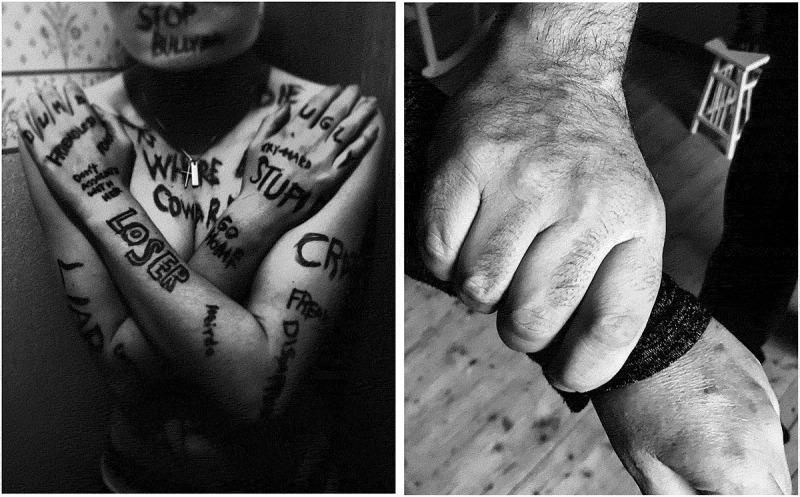
Examples of photographs taken by participants, illustrating violence.

The rural school with which we collaborated is the only upper secondary school in the region. Some students live in the area, but the majority of students live in the surrounding rural areas and commute daily. Participants were divided into small groups ranging from six to eight girls. Each group met for five workshops over a period of six weeks (on average). Each workshop, framed by photovoice, lasted between 60 and 180 minutes. Ahead of every workshop, the girls were prompted to take 3 to 5 pictures to display, share, and discuss in a group. The pictures were sent to the first and third author, who printed the photographs and facilitated the workshops. During the workshops, each girl presented their photos one at a time and what it meant before the group could comment and ask questions. Figure 1 show example of two photos taken by participants illustrating violence based on the prompt “what are the different forms of violence against girls in your school or community”. All conversations were audio-recorded using a digital voice recorder and transcribed verbatim. Any information that could indicate the participant’s identity was removed. The results presented in this paper are from the analysis of the discussions from the workshops, hence in this way the results are not co-constructed. However the participants have been a large part of developing the themes and subthemes throughout the project and co-constructed results will be presented elsewhere. In this article, we have used photovoice for two purposes: 1) for social change and 2) as a data collection method.

In Phase 2 of the study, we scheduled a session on community mapping to enable the students to reflect on their local community’s resources, strengths and challenges. In Phase 3, we aimed to address the purpose of the study, i.e., to learn about the gendered violence facing girls in their everyday life. For the girls’ first task, which served as an introduction to the study method, they were asked to take pictures of: *feelings and/or situations of being safe*; and *feelings and/or situations of being unsafe for girls in your community*. The girls were asked to bring the pictures to the next workshop, and to elaborate on why they took the photo and what it represented to them. To indicate feelings of safety, participants took pictures of items and spaces such as their front door, their own room, and details of interiors. Pictures of items and situations such as liquor bottles, dark tunnels, and being followed by men represented feelings of being unsafe. All workshops were based on the photographs, and the dialogues were partly guided by a question strategy developed by (Wallerstein & Bernstein, 1988) called SHOWeD tool; *What do you see here? What is really happening here? How does this relate to our lives? Why does this situation exist? What can we do about it?*.

In Phase 4 of the study, the workshops focused on the questions: *what are the different forms of violence against girls in your school or community?* and *what are the consequences of such violence?* The girls took photographs of, for example, bullying, groping, bruises, and online harassment to represent different forms of violence. The consequences of the represented violence were partly shown through photographs of self-harm, mental illness, failing school, and suicide. During the workshops we continuously returned to the importance of privacy and respect with regards to the images, to avoid reproducing stereotyped narratives.

The third part of photovoice, which is to reach policy makers is included in the overall project, and throughout the project data has been utilized in many different ways. Together with the girls, we have created and photo exhibition that has been recognized by the county government and widely presented. The different strategies and ways to reach policy makers is however not included in this paper, but will be elaborated on elsewhere.

### Data analysis

Data was analysed using inductive and deductive thematic analysis, a flexible method suitable for condensing and organizing large amounts of data and for developing themes found within particular research questions (Braun & Clarke, 2006). The analysis started with a deductive approach based on the specific issues raised by the prompts. Due to the participatory nature of photovoice, the girls themselves played a large part of developing and categorizing data in this phase. As new data emerged from the material through workshop discussions, an inductive approach was applied. Discussions about the prompts and the photographs showed that gender intersects with existing values on how to act, look, and behave. Discussions on how the girls in the study perceive their environment, activities and interactions has made a large contribution to the transition from a deductive to an inductive approach.

Workshops and conversations, combined with early observations and personal notes, catered for the first stage, *(1) familiarizing yourself with your data*. Data transcription was carried out by the first author, and this process played a significant part in getting familiar with the data. While reading through the material repeatedly to obtain a sense of the meaning of the text as a whole, key words and reflections were written down in order to discern recurring themes. The coding process, *(2) generating initial codes*, started with the categorization of themes discussed during workshops related to the prompts presented in Phases 3 and 4 under *Process and Procedure*. Initial coding resulted in codes such as “afraid of being judged” or “feeling insecure”. During the next step,*(3) searching for themes*, quotes and sentences were extracted and grouped according to repeated themes which related to the aim of the study. The coding was influenced by the existing theories of Butler (2004, 1990) and Connell and Messerschmidt (2005). While some initial codes were merely renamed, other initial codes were merged and formed new themes. The next stage, *(4) reviewing themes*, entailed revisiting all selected quotes and extracts to ensure coherence and connections. The final stage, *(5) defining and naming themes*, acted as a control to ensure relevance to the aim of the study. For example “afraid of being judged” subsequently became “label”, and “appearance and performance” became “doing gender”. At the end of this procedure, following further changes, two main themes and four sub-themes were created. Procedure, structure of themes, and credibility checks was frequently discussed between the authors.

### Ethical considerations

In Phase 1 of the study, students and teachers were given a presentation about the project detailing its aims and concerns, followed by ethical instructions about anonymity and confidentiality. Information was given in verbal and written format, along with photo ethics, to ensure that potential participants had an appropriate understanding of the procedure and purpose of the study. This process also ensured that they were aware of their right to withdraw from participation without negative consequences. Students wishing to take part then signed a written consent form. The project was ethically approved by the Regional Ethical Review Board at Umeå University as being in accordance with ethical standards, dnr 2017/58-31

## Results

In this section of the paper, we describe two main themes that arose in the data and present the results in relation to the study’s aim, supporting our interpretations with quotes from the data. We provide further interpretations in the discussion. The first theme, *constant fear*, is divided in two sub-themes that explain what type of fear the girls expressed, and how this fear is entangled in their everyday lives and marginalizes girls’ social space. The second theme, *strategies*, focus on the strategies that girls employ to manage their constant fear. This theme is also divided, in two sub-themes, with a discussion of how gender norms inform behaviour and expectations. We used pseudonyms to protect the participants’ anonymity.

## Constant fear

Being exposed to gendered violence, subtle expressions of gendered power such as gazes and rumours, and calculating possible risks linked to this several times a day, can all contribute to the building up of a constant state of fear. The concept of fear was central in discussions; more specifically, the girls’ fear of men and of rape. The participants also expressed a constant awareness of how their behaviour directly influenced how they were perceived by others. Two main themes relating to fear were identified, as presented below:

### Fear of being raped

The perceived threat which emerged as most prominent was men; men in groups, drunk men, being alone outside in dark/deserted places during late hours. The girls expressed a general fear of not knowing who is hiding in the dark, “ready to physically attack and rape”. The fear that the girls talked about also included other threats such as being held against their will, and being hit, but rape was the most distinct fear to emerge from our discussions. The girls discussed an incident that allegedly happened in another upper secondary school where one of them, Sandra, was previously a student. The other members of the group were familiar with the incident through rumours. A girl had been raped by an older boy (who was not a student) during school hours, and the group discussed the damage that this had caused:

Ann: A girl was raped in a toilet cubicle at school, everybody knew, there were lots of evidence, she was thirteen and he was twenty. He got sentenced to two months. (…) He ruined her life, and all he gets is two months! Where is the justice in that?

Sandra: We met that guy two months after he was released and he was living life, and we all went: “What is he doing here, this is really scary”.

A frustration was apparent among the girls regarding lack of support for the violated girl, as well as the insecurity and fear it caused other girls at the school. In contrast, they could not see that the perpetrator’s life was affected at all. Several girls expressed anger linked to the probability of the girl being blamed for being raped, as well as being considered disgusting and dirty by others.

### Fear of being labelled and excluded

When discussing physical and psychological violence, the girls agreed that it is the latter that never heals; bruises fade, but the psychological damage will always remain. The girls expressed the opinion that being exposed to psychological violence such as online harassment, rumours, and name-calling can lead to a great level of insecurity and a skewed self-image:

Olivia: You get really insecure and go, “My god, am I like that?”

Mia: You never know what people really think of you personally, so when they say “You are so ugly, you whore”, you start to question yourself, what if I am, kind of …

Olivia: Yes, then you start thinking about it the whole time, and in the end you start believing it.

There are visible gender norms, informed by patriarchal structures, which are attached to heteronormative ideas and attitudes among the girls in relation to performativity. These norms affect daily concerns about physical appearance, and these concerns are visible in how the girls discuss and develop safety strategies through appearance:

Sandra: (…) You may feel uncomfortable if you wear a certain kind of outfit. A skirt can make you feel vulnerable, you can feel vulnerable [unsafe] if you wear your hair tied up or loose, if you wear makeup or not …

Interviewer: Why does your hairstyle affect your feeling of being unsafe?

Sandra: I don’t know … you care what people think.

Gender norms are also visible when listening to the girls’ conversations about how women in general behave under the influence of alcohol. Maria says: “Many girls, I don’t know, it might sound vicious, but they throw themselves at boys and act like whores”. Alva reacts to this comment and asks, with a firm tone, what she means by that. Maria again links this behaviour to being a “whore” before adding that she, personally, rejects such attitudes as wrong. She speculates whether these attitudes, in other girls, might be rooted in jealousy. During further discussions about how to behave and dress, online harassment, receiving “dick pics” (where boys and men send unwanted pictures of their own, or others’, erect genitalia via cellphone) and how to handle unwanted attention, the girls express a fear of being either an outsider or labelled a “slut”.

Stina: (…) if you say no, I mean resist the one that is touching you because you don’t want to be touched, you stand the risk of being an outsider.

Interviewer: So if you resist unwanted touching, you risk being excluded?

Stina: Exactly.

The quotes above reveal that there is an awareness of possible consequences of putting up resistance, and these contradictory positions are visible when the girls talk about how to handle unwanted attention from boys. The girls continued to talk about resistance and gave the example of an online scenario where a girl receives a text from a boy saying “send nudes”. If she agrees, she risk being labelled a “slut” or “easy”, but if she resists, she risks being excluded and labelled as a “prude” or “boring”.

## Strategies

During the discussions it became evident how the girls apply different strategies to avoid risks and how these strategies are related to the constant state of fear. Experiencing fear when walking alone at night was common, and some of the mentioned strategies were holding keys in their hands, owning a legally approved self-defence spray, avoid walking alone late hours or call someone while walking, avoid certain public spaces and walk with confidence. In conversations about this, several girls nodded their heads in recognition at descriptions of strategies to avoid fear.

Denise: I try to always have someone walking home with me.

Clara: Sometimes you run home, just because you’re scared that something can happen.

Even though fear of men and rape surfaced as the most prominent among the girls, general fear is also something that relates to how one is viewed by others. In the material we found two main strategies directly linked to fear, as presented below:

### Being “appropriately” sexually active

To understand this strategy, we begin with hegemonic norms linked to sexuality (i.e., norms that assign an accepted behaviour to a specific gender). Girls in all groups expressed the view that girls need to act according to different gender expectations, compared to the boys. This category intertwines with the fear of being labelled as girls’ sexuality is constrained by the risk of being labelled a “whore”, and girls need to restrict both their sexuality and their number of sexual partners to avoid this label:

Maria: If a girl has sex with someone who’s not her boyfriend, or if she has had sex with, say, two people, then you are [seen as] a whore, but guys can have sex with as many as they like. It’s kind of something to brag about.

Sara: They can sit and just say “Oh, I’ve had sex with (NN)”, like I care? It’s so damn stupid. Like it’s really cool. Imagine if a girl said stuff like that, then it would be like “What the hell is wrong with her?” kind of.

Maria: Girls are not allowed to show their sexuality or anything, they must be nice and kind and not talk dirty, but guys are allowed to talk about everything.

Leah: They [boys] can do what they want (…) they can break up with girls and get new ones (…) They can do whatever they want, so … they do everything.

The above quotes, and the girls’ further discussions related to their own sexuality, show an awareness of how status is monitored and linked to relationship and being someone’s girlfriend. It is acceptable for girls to be sexually active as long as they have a boyfriend, but even a relationship comes with limitations:

Denise: You can’t change partner too often, you need to stay together for a long time (…) and you can’t have sex if you’ve been together for only a week.

In the conversations above, categorizations of gender are visible in terms of how the girls understand society’s view on boys and girls. They are also visible in how the girls position themselves within these discussions of sexuality.

### Appearance and performance for increased feelings of safety

Girls are highly aware of the restricted spaces they occupy, and many of them continually try to guard their current space (or push boundaries) through various strategies. Calculations of vulnerability and safety go hand-in-hand with daily choices, and the girls have found numerous strategies to resist feelings of being unsafe. These include “modifying their clothes and other aspects of their appearance and restricting their activities to reduce their perceived risk of violence, thus limiting their use of public space” (Hollander, 2001, p. 105). The girls discussed some of their applied strategies to promote increased feelings of safety, which included acting confidently, standing up straight, walking fast, or calling someone:

Clara: You arm up, you prepare. I walk differently, I walk determinedly so they can see that “don’t mess with me”.

Interviewer: So you have a strategy?

Clara: Yes, my mom has told me what to do when being out. “You should walk with sureness so they can see that you are confident. Look down, do not seek eye contact”.

Such strategies might be so integrated into daily routines that they are no longer considered to be specific safety strategies among the girls themselves. As an example, one of the girls said that their group of friends always felt safe, so she could not relate to the notion of strategies at all. To demonstrate what she meant, she said that if (for example) one girl in the group needed the bathroom while in a public space, they made sure that she was accompanied by at least one other friend. They never left anyone in the group alone or behind when walking home; therefore, they felt safe. When asked why she and her friends found it necessary to look after each other, she appeared very surprised. It seemed as if it had never occurred to her that what they were doing were, in fact, strategies to promote increased feeling of safety.

## Discussion

Our findings consisted of two main themes: *constant fear* and *the strategies girls employ against the fear*; and four sub-themes: *fear of being raped, fear of being labelled and excluded, being appropriately sexually active*, and *appearance and performance for increased feeling of safety*. These findings contribute to the understanding of the challenges facing young girls in contemporary Sweden and highlight the importance of continuing to discuss gender related topics. The results show that girls’ experience of gendered violence is enmeshed in their everyday life, and they are aware that different rules apply to them, compared to boys. Our perception of the themes and results, based on the girls’ insights, is that the violence facing girls is made possible through performances of gender (Butler, 1990, 2004), largely regulated by hegemonic masculinities (Connell & Messerschmidt, 2005) which control girls’ actions and social space. Situations linked to gendered violence are related to constant negotiations of self-worth, safety, and wellbeing, entangled in fear and safety strategies. This article draws attention to how adolescent girls themselves understand and navigate their “everyday”. Concepts of femininities and masculinities are influenced and regulated by performativity (Butler, 1990, 2004) and the hegemonic notion of masculinity (Connell & Messerschmidt, 2005). There are dominant attitudes on how to behave in accordance with one’s gender, and listening to the girls’ discussions reveals inequalities in social spaces that are favourable to boys. However, even though girls see and resist this gender hierarchy, they also unintentionally follow the existing patterns in society.

Gendered violence functions as a form of social control, affecting girls largely through fear regardless of whether or not they have personally experienced violation (Wendt, 2002). This is also visible in the report from The *Swedish* National *Council* for *Crime Prevention*, (Axell, 2018) which indicates that one-third of women in Sweden do not feel safe going out late at night. Fear of being raped is part of the lived experience of being a woman, and can be described as an extra burden entangled in a constant awareness of “what if” and the presence of possible danger. This was recognized as a problem several decades ago (Gordon & Riger, 1991). Women’s and girls’ general fear of being raped differs from fear of other physical attacks such as being robbed, because rape is interwoven with gender-specific shame narratives that hold women responsible for not being able to avoid being victimized (Gordon & Riger, 1991; Weiss, 2010). Understanding girls’ general perception of fear is central to understanding gendered inequalities within social arenas. The girls’ discussion about fear of rape, through the incident that allegedly happened in another upper secondary school, reveals how incidents and narratives can underpin fear, even if individuals have not personally experienced being physically violated. This is partly because of regular conversations about danger and female vulnerability (Griffin, 2015; Hollander, 2001) and partly because girls and women are more or less taught to fear rape through the media and movies, along with distrust with the legal system (Gordon & Riger, 1991). Approaching the discussions from a gendered perspective, through Butler’s (2004, 1990) notion of patterns of dominance and subordination, shows that the girls’ understanding of men as a source of threat and the main cause of girls’ fear is reinforced, as is the understanding that different rules apply to boys and girls. Despite the participants’ awareness that this is wrong, it contributes to ongoing constructions of gender that place men and boys in a position of power over women and girls (Connell & Messerschmidt, 2005).

Butler argues that gender is created and recreated through imitations of imitations that continuously are performed (Butler, 1990). Conversations about female sexuality reveal several layers of ongoing gender constructions, performativity, and contradictory positions. The girls unintentionally uphold patriarchy by positioning themselves and other girls in relation to stereotyped ideas of “appropriate” behaviour, and this is particularly visible when Maria reveals that she thinks girls who “throw themselves at boys” are “acting like whores”. It was not until she was asked to explain what she said that she actually reflected on the comment and added that the attitude applied to others, not her. Similar results are evident in a study with a focus on feminine and masculine ideologies, in which girls expressed frustration around contrasting principles related to their sexuality, while simultaneously degrading other girls’ sexuality if it did not conform to femininity as legitimized through a monogamous relationship (Tolman, Davis, & Bowman, 2016). Girls’ sexuality is monitored, and regardless of how the girl responds, it is likely to lead to undesirable consequences. The only option girls have of resisting what Dines refers to as being “fuckable” is to be invisible (Dines, 2010). If girls explore their sexuality, they risk being labelled a “slut”, but if they do not, they risk being labelled as “uncool” and “boring”. Dines (2010) states that these contradictory positions (and girls’ attempted strategies to navigate through unwanted attention from boys) reveal a “damned if you do, damned if you don’t” situation that stretches far beyond sexuality. Since there are no direct parallel labels or risks for boys who conforms to heterosexual attitudes and behaviours, there are fewer restrictions on their sexuality. This creates a marginalized space for girls, and this restrictions provide a larger space for boys (Jeffner, 1998).

Another sign of conflicting gender norms among the girls is visible in the discussion of “dick pics”. Despite an overall perception from a number of girls that both receiving “dick pics” and being exposed to unwanted touching largely happens to only a “certain kind” of girl, further discussion revealed that almost every girl in school had had this experience. This reveals that while the girls actively resist and reject labels, they unconsciously position themselves accordingly to the same structures. Despite the girls’ awareness of how wrong gendered ideas and performances are, and of how the boys largely determine the girls’ position, they are still part of “doing gender” (West & Zimmerman, 1987) when positioning themselves within stereotyped ideas linked to femininity and expected behaviour (Connell, 2003). A fear of being labelled as “that kind of girl” intersects with discourses about heterosexuality, body, and performances, and the girls continuously balance possible outcomes attached to behaviour.

Constructions of gender within the social arena are also visible in the school context. As numerous scholars have argued (and as our data suggest) there are expectations linked to gender in this area, in the sense that girls’ voices and actions are more strictly controlled than boys’ (Gordon, 2006; Gordon, Holland, Lahelma, & Thomson, 2008). Inaccurate ideas of boys as more adventurous, loud, and rebellious than girls allow boys to behave differently. There are regulations on both appearance and behaviour for girls, without there necessarily being similar expectations of boys (Einberg, Lidell, & Clausson, 2015; Gordon et al., 2008). These normative ideas not only serve as an excuse for boys’ actions but also largely place responsibility on girls. Furthermore, girls are the ones expected to deal with sexual harassment, while the perpetrators’ (i.e., the boys’) actions are largely overlooked (Robinson, 2005). While “boys will be boys”, girls are expected to draw the line or be “mature enough” to handle unwanted attention, and these expectations on girls start at an early age (Gillander Gådin, 2012). During conversations related to how boys are allowed to behave in a more rebellious way, the girls expressed frustration. However, there was also a sense of acceptance of the situation and an attitude of “it is just the way things are and always have been”. The girls unconsciously took responsibility for handling different situations, or found mitigating excuses for boys’ behaviour, on the basis of them not fully understanding the consequences of their actions. While norms about gender assign responsibility and virtue to femininity (Skeggs, 2001) the same norms assign contrasting traits, such as exerting power over others and avoid showing vulnerability, to masculinity (Jeffner, 1998).

### Methodological considerations

#### Strengths & limitations

The study contributes to Swedish studies focusing on rurality and emphasizes that gendered violence and fear have similar patterns among young people, regardless of geographical position. It is possible that the results could have been different if data was collected in an urban environment, but we have little reason to believe this would be the case. On the contrary, we suggest that gendered violence among Swedish young people has similar characteristics regardless of geographical location. The strength of this qualitative study partly lies in the interactional approach of photovoice as a method. We claim, in line with (Harper, 2002), that using photos allows us to gather more information than traditional focus group interviews, because the discussions of the photos and the feelings they evoke gives information that is both richer and qualitatively different. When the girls visualized how they perceived their environment, activities, and interactions, this provided valuable insight into the school environment and the community at large. Simultaneously, it made the girls aware of normalized inequalities. The method allows us to access insights and viewpoints that we otherwise might have neglected. With the use of cellphone cameras, the respondents were able to work without our presence, which may have been perceived as less intrusive to them than if we had been there (Ahrne & Svensson, 2011; Wang & Burris, 1997).

Although this study provide valuable insights, some limitations should be noted. Despite the advantages of using photovoice, it also has some shortcomings. It is time-consuming both for participants and researchers, and requires preparation with photo ethics before starting, as well as setting aside time for participation in group discussions and workshops during the project. While we repeatedly emphasized that the focus is on women’s general situations and not on personal experiences, it is possible that the sensitive topic caused discomfort for some of the girls. Furthermore, there is a possibility that any existing social hierarchies within the groups may have silenced some girls, who might have shared more of their insights in a different context such as private conversations.

## Implications

Masculinity is often confused with, and associated with, domination and violence. By actively discussing these structures from an early age, and by allowing boys to aspire to non-violent, non-dominant forms of masculinity, we could replace patriarchy with a system of equal human rights and gender equality. Working for gender equality should be central to every school development plan, in order to promote increased safety, health and wellbeing among young people. It should also be emphasized that even though the perspective of girls is central to this study, the responsibility to prevent violence and change the situation does not rest on them. Instead knowledge produced together with the girls should be used to inform policy makers.

In order to prevent boy’s and men’s violence against girls and women, there is a need to extend the understanding of the gendered and social norms that uphold such violence. Even if there are few evidence-based programs preventing violence against girls and women, those who have showed a positive effect have some common features, e.g., being participatory, engaging stakeholders from different sectors and support a critical discussion of gender. A wide range of arenas, both public and private needs to be involved on multiple levels to prevent and address gender based violence (Ellsberg et al., 2015).

## Conclusion

In this article, we discuss and analyse data from 35 girls studying in an upper secondary school in a rural part of Sweden. While the girls differ in age, study program, and ethnicity, they are bound together by their common understanding of fear, safety strategies, and the importance of appearance. We found that hegemonic masculinity and performances strongly influence language and behaviour among young people, both in a school context and during leisure time. Despite Sweden ranking highly for gender equality, there are several indications of this not permeating throughout society. Our study shows that some girls in Sweden are in a situation where they need to apply strategies to cope with fear and harassment on a daily basis. Our study cannot be used to assess the extent of violence and harassment against girls. However, knowing about the global burden of violence against girls and women, including Sweden, we assume that our results are transferable to other places as well, both national and international.

There is an urgent need for more research concerning gendered violence among young people within schools and other arenas. Girls are marginalized in a range of situations and interactions, and the social spaces that girls occupy are significantly smaller compared to the social space belonging to boys. This is reflected in how the girls position themselves in gender-related discussions, and in how they understand being treated by society at large. Our interpretation is that women’s and girls’ experiences of exposure to gendered violence, fear, and applied strategies both derive from existing gender inequality and contribute to this inequality by continually reproducing it. The deconstruction of existing discourses linked to hegemonic masculinity and gender performances among young people should be prioritized from an early age. Such discourses not only limit and control girls’ social space, but also inform boys’ behaviour and undermine less aggressive forms of masculinity.
